# Reduced cardiac function is associated with cardiac injury and mortality risk in hospitalized COVID‐19 Patients

**DOI:** 10.1002/clc.23479

**Published:** 2020-10-14

**Authors:** Lu Q. Chen, Joseph Burdowski, Ravi Marfatia, Jonathan Weber, Kathleen Gliganic, Nancy Diaz, Neiman Ramjattan, Haoyi Zheng, Dennis Mihalatos, Lin Wang, Eddy Barasch, Amanda Leung, Aasha Gopal, Jason Craft, Xiaoli Ren, Kathleen Stergiopoulos, Allen Jeremias, George Petrossian, Newell Robinson, Joseph Levine, Richard A. Shlofmitz, Ronald J. Gulotta, Stefan M. Muehlbauer, Charles L. Lucore, J. Jane Cao

**Affiliations:** ^1^ Department of Research and Department of Cardiology St Francis Hospital, The Heart Center Roslyn New York USA

**Keywords:** cardiac function, COVID‐19, mortality, SARS‐CoV‐2, troponin

## Abstract

**Background:**

Cardiac injury is common in COVID‐19 patients and is associated with increased mortality. However, it remains unclear if reduced cardiac function is associated with cardiac injury, and additionally if mortality risk is increased among those with reduced cardiac function in COVID‐19 patients.

**Hypothesis:**

The aim of this study was to assess cardiac function among COVID‐19 patients with and without biomarkers of cardiac injury and to determine the mortality risk associated with reduced cardiac function.

**Methods/Results:**

This retrospective cohort study analyzed 143 consecutive COVID‐19 patients who had an echocardiogram during hospitalization between March 1, 2020 and May 5, 2020. The mean age was 67 ± 16 years. Cardiac troponin‐I was available in 131 patients and an increased value (>0.03 ng/dL) was found in 59 patients (45%). Reduced cardiac function, which included reduced left or right ventricular systolic function, was found in 40 patients (28%). Reduced cardiac function was found in 18% of patients without troponin‐I elevation, 42% with mild troponin increase (0.04‐5.00 ng/dL) and 67% with significant troponin increase (>5 ng/dL). Reduced cardiac function was also present in more than half of the patients on mechanical ventilation or those deceased. The in‐hospital mortality of this cohort was 28% (N = 40). Using logistic regression analysis, we found that reduced cardiac function was associated with increased mortality with adjusted odds ratio (95% confidence interval) of 2.65 (1.18 to 5.96).

**Conclusions:**

Reduced cardiac function is highly prevalent among hospitalized COVID‐19 patients with biomarkers of myocardial injury and is independently associated with mortality.

**Abbreviations**
CAD: coronary artery disease
LVEF: left ventricular ejection fraction
RVEF: right ventricular ejection fraction

## 1 INTRODUCTION

COVID‐19 is caused by infection with severe acute respiratory syndrome coronavirus 2 (SARS‐CoV‐2). As of October 1, 2020, more than 34 million people worldwide became infected and over 1 million have died. The complex pathophysiology of COVID‐19, which includes cell death due to intracellular multiplication of virus, hypercoagulability, and cytokine storm^1^, ^2^, ^3^, ^4^ contributes to a wide array of complications including acute respiratory distress syndrome, multi‐organ failure, sepsis, disseminated intravascular coagulation, as well as complex cardiac injury.^5^, ^6^, ^7^, ^8^, ^9^, ^10^, ^11^ Many cardiac features have been reported including high prevalence of cardiac troponin elevation due to myocarditis or acute coronary syndrome, pericardial effusion and cardiac thromboembolism.^9^, ^12^, ^13^, ^14^, ^15^, ^16^, ^17^ Troponin elevation in COVID‐19 patients has been linked to adverse clinical outcomes.^12^ Additionally, abnormal cardiac function by echocardiography has been reported,^18^, ^19^, ^20^ and is associated with biomarkers of cardiac injury.^21^, ^22^ It remains largely unknown if reduced cardiac function is associated with biomarkers of cardiac injury and with adverse clinical outcomes. In this retrospective study we aim to examine cardiac function by echocardiography in hospitalized patients with laboratory‐confirmed SARS‐CoV‐2 infection, with and without cardiac injury, and associations with mortality.

## 2 METHODS

We performed a cohort study involving consecutive, laboratory confirmed COVID‐19 patients admitted to St. Francis Hospital, Roslyn, NY who underwent echocardiogram between March 1, 2020 and May 5, 2020. A waiver for individual consent was approved by the Institutional Review Board to allow for retrospective case review. Patients' demographic data, clinical diagnoses, vital signs, laboratory results, electrocardiograms, echocardiogram and chest radiograph or chest computerized tomography were retrospectively collected from electronic medical records.

All patients had a laboratory confirmed COVID‐19 diagnosis using the reverse transcriptase polymerase chain reaction (RT‐PCR) test (Cobas SARS‐CoV‐2, Roche, Indiana, USA and the Xpert Xpress SARS‐CoV‐2, Cepheid, California). Unless otherwise specified, laboratory values are expressed continuously. When serial cardiac troponin‐I values were reported the peak value was selected for the analysis. The upper normal limit for cardiac troponin‐I was >0.03 ng/mL in our institution (Abbott Architect system for STAT troponin‐I, analytic sensitivity ≤0.01 ng/mL). Troponin‐I cutoffs of 0.04 to 5.00, and > 5.00 were also used for evaluation.

### 2.1 Echocardiograph

Echocardiograms were performed using Philips IE 33, Philips EPIQ 7 (Philips Healthcare, Andover MA), or GE VIVID S70 (GE Healthcare, Chicago, IL) scanners. Due to the highly contagious nature of SARS‐CoV‐2 infection, 54 (38%) studies were performed as limited exams, which included evaluation of biventricular function, color Doppler of the valves and tricuspid velocity when present and pericardial effusion. There was one case where image quality was limited for the evaluation of cardiac function although it was adequate for the assessment of pericardial effusion. Left and right ventricular systolic function were evaluated qualitatively. Left and right ventricular (RV) ejection fractions (EF) were estimated visually and considered reduced when <50%.

The RV systolic pressure was estimated based on the velocity of tricuspid regurgitation jet if present using the simplified Bernoulli equation and adding the estimated right atrial pressure, which was assumed to be 8 mmHg if the inferior vena cava was not well visualized. There were 59 (41%) cases where tricuspid regurgitant velocity was assessed. Pulmonary hypertension was defined as having RV systolic pressure ≥ 35 mmHg with mild, moderate and severe disease corresponding to pressure 35 to 44 mmHg, 45 to 59 mmHg and ≥ 60 mmHg. Significant valvular abnormalities consisted of valvular stenosis or regurgitation ≥ moderate (2) in severity.

### 2.2 Statistical analysis

We examined associations between a diagnosis of abnormal cardiac function, clinical laboratory results and all‐cause mortality in the context of COVID‐19. Laboratory values, events, and diagnostic counts are presented as mean (SD), median (interquartile range) or frequency (percent) as appropriate for data type and distribution. Stratified analysis of patients with a past medical history of CAD (defined as history of myocardial infarction, percutaneous intervention with coronary stenting, or coronary artery bypass grafting) was conducted using *χ* ^2^ tests. We present odds ratios of reduced cardiac function and significant laboratory findings (with 95% confidence limits) associated with all‐cause in‐hospital mortality from unadjusted logistic regression models as well as adjusted models (including age, gender, and history of CAD as covariates). All analyses were performed using SAS version 9.4 (SAS, Inc., Cary, NC).

## 3 RESULTS

Between March 1, 2020 and May 5, 2020 there were 843 COVID‐19 cases hospitalized in St Francis Hospital, Roslyn, NY. Of those, 143 consecutive patients underwent echocardiography, and were selected as the subjects of the current study. The mean age was 67 ± 16 years, and 62% (N = 89) were male. The most common comorbidities were hypertension (69%) and CAD (30%) (Table 1). Evidence of pneumonia was found in 73% (N = 105) of the cases either by chest radiograph or CT. Troponin‐I was tested in the majority of patients (92%, N = 131) with 59 (45%) patients having elevated troponin level, which was more common in patients with history of CAD than those without (62% vs 38%, *P* = .014). An ST‐T wave abnormality was found in 71 (50%) of 141 patients who had a 12‐lead ECG on file. It was also more prevalent in patients with CAD than those without (72% vs 41%, *P* < .001).

**TABLE 1:** Clinical, laboratory, and echocardiographic findings

	N (%), mean (std), median (IQR)
Age (years)	67 (16)
Male	89 (62)
Body mass index (km/m2)	29.0 (6.3)
Ever smoking	52 (36)
Comorbidities	
Hypertension	99 (69)
Diabetes	54 (38)
Hyperlipidemia	80 (56)
Coronary artery disease	43 (30)
Stroke	15 (10)
Pulmonary disease	20 (14)
Chronic renal insufficiency	27 (19)
Cancer	23 (16)
Vital signs	
Heart rate (BPM)	83 (21)
Blood pressure, systolic (mmHg)	122 (23)
Blood pressure, diastolic (mmHg)	69 (14)
Max WBC (x 10̂3 /mcL)	12.2 (7.8‐19.4)
Hematocrit (%)	39.0 (6.1)
Hemoglobin (g/dL)	13.1 (11.1‐14.4)
Max platelet count (x 10̂3 /mcL)	328 (153)
BUN (mg/dL)	19 (13‐28)
Creatinine (mg/dL)	1.0 (0.8‐1.5)
Alkaline Phosphatase (U/L)	77 (64‐105)
AST(U/L)	39 (27‐67)
ALT (U/L)	33 (20‐60)
Peak Lactic acid (mmol/L)	2.3 (1.5‐3.4)
LDH (U/L)	564 (402‐860)
Prothrombin time (second)	11.3 (10.8‐12.2)
Partial thromboplastin time (second)	32 (30‐35)
International normalized ratio	1.1 (1.0‐1.2)
D‐dimer (mcg/mL)	3.6 (1.2‐9.7)
Peak C‐reactive protein (mg/L)	147 (75‐180)
Ferritin (ng/mL)	854 (341‐2292)
Procalcitonin (ng/mL)	0.16 (0.09‐1.07)
Troponin >0.03 ng/dL	59 (45)
Troponin 0.04 to <1.0 ng/dL	43 (33)
Troponin >1.0 and ≤ 5.0 ng/dL	7 (5)
Troponin >5 ng/dL	9 (7)
b‐type natriuretic peptide >400 pg/mL	19 (23)
12‐lead ECG	
Normal sinus rhythm	96 (67)
Sinus tachycardia	14 (10)
ST‐T wave abnormality	71 (50)
among whom troponin elevation present	32 (52)
among whom troponin elevation absent	30 (48)
Atrial fibrillation	19 (13)
Paced rhythm	3 (3)
ST‐T wave abnormality OR troponin >0.03	88 (68)
Chest X‐ray or chest CT	
Bilateral Pneumonia	88 (63)
Unilateral pneumonia	17 (12)
No pneumonia	34 (24)
Pleural effusion	19 (14)
Clinical outcomes	
Elevated Tn or ST‐T wave abnormality	88 (68)
New onset acute coronary syndrome	7 (5)
New onset heart failure	23 (16)
New onset of atrial fibrillation	13 (9)
Ventricular tachycardia	2 (1)
Cardiac tamponade	5 (4)
Pulmonary embolism	11 (8)
Stroke or transient ischemic attack	6 (4)
Acute kidney injury	39 (27)
Mechanical respiratory support (intubation or BiPAP^a^)	50 (35)
among whom cardiac echo conducted while on support	32 (64)
Ventilator	39 (27)
BiPAP	11 (8)
Hospital length of stay, days (n = 125 discharged patients)	9 (9)
All‐cause mortality	40 (28)
Echocardiographic findings	
Left ventricular ejection fraction (%)	55 (50‐60)
Reduced left ventricular systolic function	34 (24)
Left ventricular ejection fraction 36% to 49%	8 (6)
Left ventricular ejection fraction ≤35%	24 (17)
Dilated left ventricle	10 (7)
Left ventricular thrombus	2 (1)
Reduced right ventricular systolic function	25 (17)
Dilated right ventricle	14 (10)
Significant valvular abnormalities	27 (22)
Aortic stenosis	8 (9)
Aortic regurgitation	3 (3)
Mitral regurgitation	12 (10)
Tricuspid regurgitation	11 (9)
Pulmonary regurgitation	2 (3)
Right ventricular systolic pressure (mmHg)	33 (27‐43)
Pulmonary hypertension	23 (16)
Mild	10 (17)
Moderate	9 (15)
Severe	4 (7)
Pericardial effusion	35 (24)
Trivial‐Small	28 (20)
Small/moderate – moderate	5 (3)
Moderate/large – large	2 (1)
Tamponade	5 (3)

^a^ Bilevel positive airway pressure.

**FIGURE 1:**
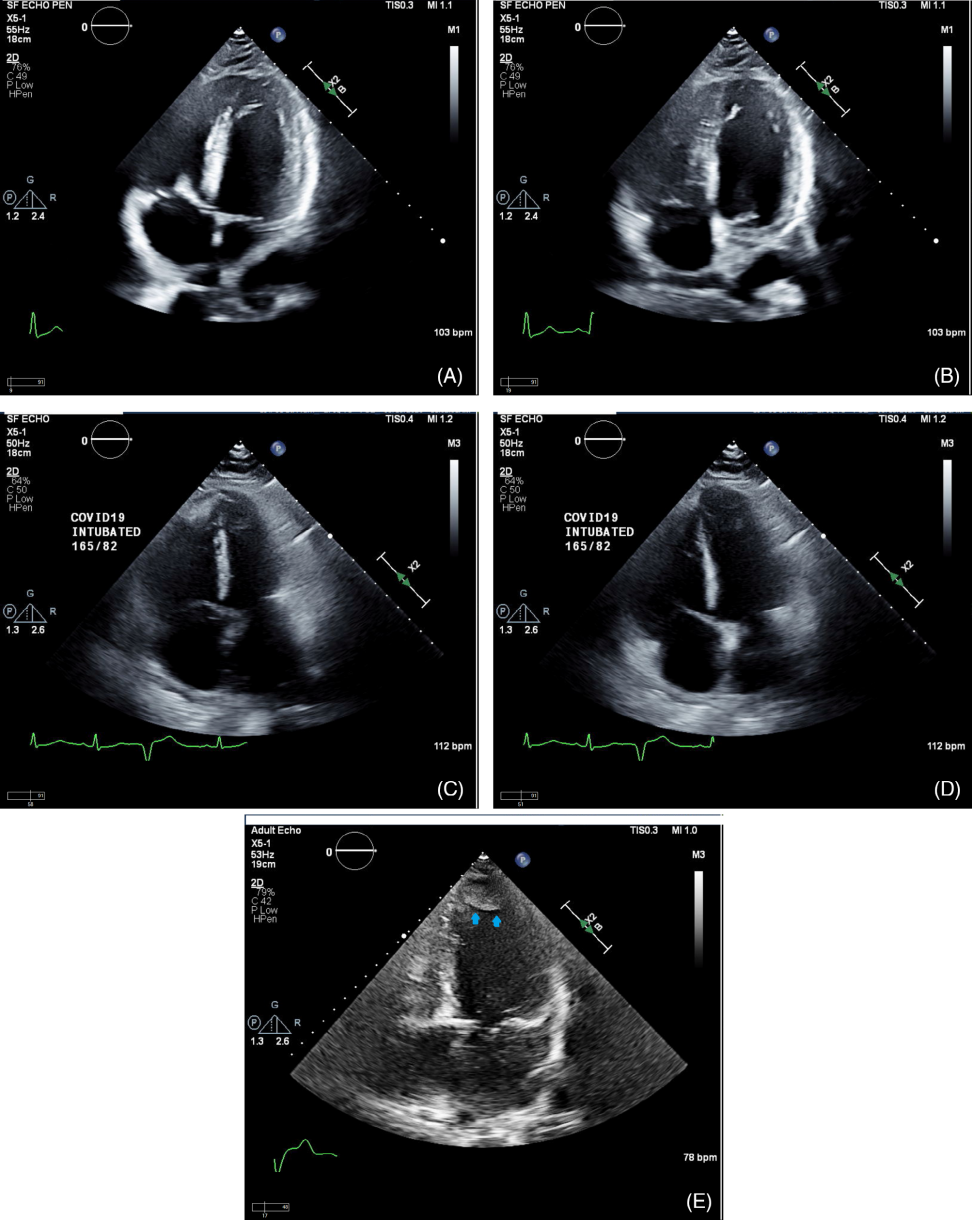
Examples of echocardiographic findings in COVID‐19 patients: severely reduced left ventricular systolic function with diffuse hypokinesis as shown in end systole (panel A) and in end diastole (panel B) of a 4‐chamber view in a 69 year old male with significantly elevated peak cardiac troponin I of 29.6 ng/mL who deceased 36 days after the admission; severely dilated right ventricle with reduced right ventricular systolic function as shown in end systole (panel C) and in end diastole (panel D) of a 4‐chamber view in a 48 year old male with mildly elevated Troponin‐I of 0.15 ng/mL, who was supported by ventilator due to acute respiratory disease syndrome; large left ventricular apical thrombus (arrows) as shown in a 4‐chamber view (panel E) in a 73 year old male patient with reduced left ventricular systolic function and mildly elevated Troponin‐I of 0.13 ng/mL

The most common indications for echo were shortness of breath (N = 37, 26%), chest pain or suspected acute coronary syndrome (N = 26, 18%) and arrhythmia (N = 20, 14%). Reduced LVEF was present in 24% (34 of 142) of the patients. Of those, two thirds had LVEF≤35% (N = 24) (Table 1, echocardiographic findings). LV thrombus was found in two cases with reduced LVEF. Reduced RV systolic function was seen in 17% (25 of 143) of the cases. The most common valvular abnormality with at least moderate severity was mitral regurgitation (10%) followed by tricuspid regurgitation (9%). A severe valvular abnormality was rare. Pulmonary hypertension was present in 23 (39%) of 59 patients measured with one third having moderate or severe disease. A pericardial effusion was found in 35 cases (24%) with cardiac tamponade physiology seen in 5 cases (3%), which required pericardiocentesis. Examples of cases and echocardiographic findings are shown in the accompanying Figure 1.

**FIGURE 2:**
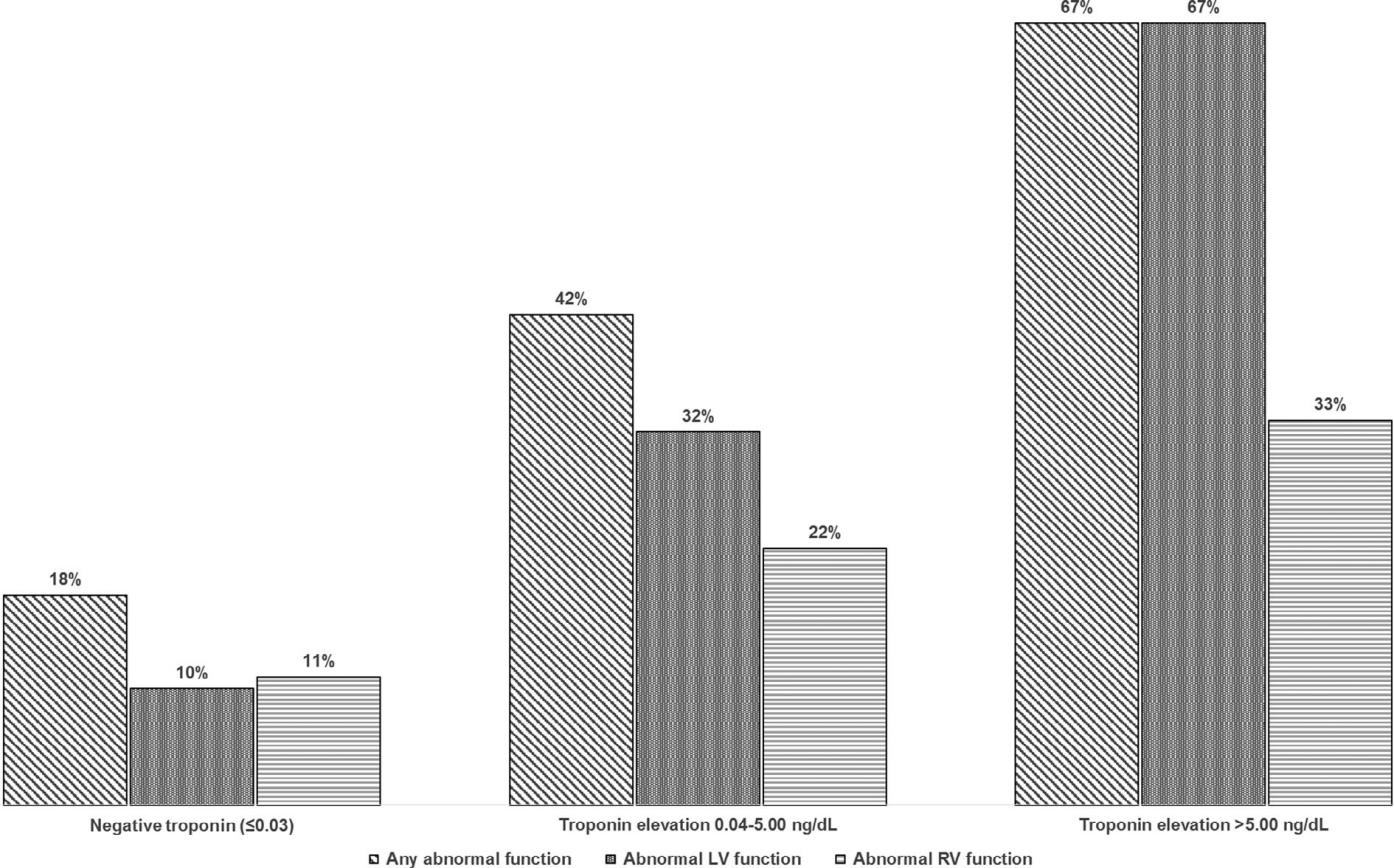
Troponin severity and abnormal cardiac function. A graded increase of prevalence of reduced cardiac function with increase of troponin‐I levels was found for patients with no troponin elevation, mild troponin increase (0.04‐5.00 ng/dL) and significant troponin increase (>5 ng/dL)

We compared the prevalence of having reduced cardiac function in subgroups (Table 2). There was a graded increase in the prevalence of reduced cardiac function of 18%, 42%, and 67% **(**Figure 2 **)** for patients with no troponin elevation, mild troponin increase (0.04‐5.00 ng/dL) and significant troponin increase (>5 ng/dL), respectively. Reduced LV systolic function was more common in patients with CAD than those without (35% vs 19%, *P* = .041). The difference was not present for RV dysfunction (*P* = .23).

**TABLE 2:** Subgroup analysis

			N (%) of cardio‐pulmonary outcomes		
	N (%) of sample	N (%) Missing	Reduced function^a^	Reduced LV function	Reduced RV function
Normal troponin‐I (≤0.03 ng/dL)	72 (55)	12 (8)	13 (18)	7 (10)	8 (11)
Elevated troponin (>0.03 ng/dL)	59 (45)	12 (8)	27 (46)	22 (37)	14 (24)
Troponin elevation 0.04–5.00 ng/dL	50 (38)	12 (8)	21 (42)	16 (32)	11 (22)
Troponin elevation >5.00 ng/dL	9 (7)	12 (8)	6 (67)	6 (67)	3 (33)
Heart failure	23 (16)	0	18 (78)	17 (74)	9 (39)
Acute coronary syndrome	7 (5)	0	2 (29)	2 (29)	1 (14)
Mechanical respiratory support^b^	50 (35)	0	21 (42)	18 (36)	12 (24)
In‐hospital mortality	40 (28)	0	19 (43)	17 (43)	12 (30)

^a^ Includes reduced left ventricular (LV) or right ventricular (RV) function<50%.

^b^ Includes ventilator or bilevel positive airway pressure.

Abbreviations: LV, left ventricular; RV, right ventricular.

The most common cardiovascular outcome included congestive heart failure (16%, N = 23), new onset of arrhythmia including atrial fibrillation and ventricular tachycardia (10%, N = 15), pulmonary embolism (8%, N = 11), acute coronary syndrome (5%, N = 7) and tamponade with subsequent emergent pericardiocentesis (3%, N = 5) (Table 2). There were 50 patients (35%) who required mechanical respiratory support with 39 patients intubated. There were 40 (28%) deaths. This is proportionally higher than the rate of fatalities in all patients at our institution who died after being hospitalized with COVID‐19 during the same time period (n = 141, 17%). Reduced cardiac function was highly prevalent among patients with heart failure (18 of 23, 78%). It was also common among deceased patients (19 of 40, 43%) and patients on mechanical respiratory support (21 of 50, 42%). After adjustment for age, gender, and history of CAD, troponin and D‐dimer elevation, reduced cardiac function including reduced LV and RV function, and heart failure were independent predictors of death (Table 3).

**TABLE 3:** Associations of cardiac findings with all‐cause mortality

	Unadjusted			Adjusted^a^		
	OR	Lower limit	Upper limit	OR	Lower limit	Upper limit
Age	1.04	1.01	1.07	–		
Hypertension	1.24	0.55	2.79	–		
Diabetes	1.76	0.84	3.69	–		
History of CAD	2.55	1.18	5.51	–		
Reduced cardiac function^b^	2.68	1.25	5.75	2.65	1.18	5.96
Reduced LV function	3.74	1.66	8.44	3.68	1.54	8.84
Reduced RV function	2.97	1.22	7.24	2.88	1.12	7.40
Troponin I elevation >0.03 ng/dL	8.39	3.30	21.30	6.73	2.49	18.21
D‐dimer	1.05	1.02	1.09	1.06	1.02	1.11
Heart failure	3.58	1.43	9.00	3.27	1.22	8.77

^a^ Adjusted for age, gender, and history of coronary artery disease.

^b^ Reduced cardiac function.

Abbreviations: LV, left ventricular; RV, right ventricular.

## 4 DISCUSSION

In this retrospective review of hospitalized COVID‐19 patients we found that reduced cardiac function is prevalent among those with biomarkers of cardiac injury. In addition, it is prevalent among deceased patients and those on mechanical ventilation. Lastly, reduced LV and RV function are among the most important cardiac predictors of death in addition to older age, elevated troponin and elevated D‐dimer.

Myocardial injury marked by the increase in troponin has been observed in hospitalized COVID‐19 patients.^5^, ^9^, ^10^, ^11^, ^12^, ^14^ The reported prevalence ranged from 12% to 28%. Those with elevated troponin are more likely to be older and to have cardiovascular comorbidities such as hypertension, diabetes, and coronary artery disease.^12^ In a recent series from Wuhan China, elevated troponin was reported to be associated with significant adverse clinical outcomes including arrhythmia, the need of mechanical ventilation and death.^12^ In recent reports based on the US experience, abnormal cardiac function was linked to troponin elevation.^21^, ^22^ In this study we found that reduced cardiac function is more than twice as prevalent when troponin elevation is present compared to those with normal troponin level. More importantly, we demonstrated that reduced left or right ventricular function is independently associated with mortality in hospitalized COVID‐19 patients even after adjusting for existing coronary artery disease.

The prevalence of troponin elevation is 45% in our cohort, which is higher than previous reports.^5^, ^9^, ^10^, ^11^, ^12^, ^14^ However, our cohort consisted of a larger number of patients with CAD among whom troponin elevation is present (more than 60%), which is similar to the reported 55% in a cohort from Wuhan, China,^12^ suggesting that patients with underlying CAD are susceptible to myocardial injury likely due to ischemia from hypoxia, plaque rapture or thrombosis of coronary circulation secondary to a hypercoagulable state. Nonetheless, reduced cardiac function is not limited to CAD patients, and is persistent after recovery from acute COVID‐19 infection.^23^, ^24^

The exact cause of troponin elevation remains unknown in COVID‐19 patients. However, several proposed mechanisms appear plausible including acute coronary syndrome, myocarditis, direct cell injury from cytokine storm and thromboembolism due to hypercoagulability.^25^, ^26^ In addition, sepsis and disseminated intravascular coagulation, as results of systemic consequences of COVID‐19 infection, may also mediate cardiac injury. In post mortem biopsies, pathological findings include interstitial inflammatory infiltration and myocyte necrosis as well as micro‐thrombosis and vascular inflammation.^26^

In severe COVID‐19 cases, hypoxia and respiratory failure are common which often result in intubation and mechanical respiratory support. Unfortunately, the fatalities are extremely high among patients supported by a ventilator.^6^ While troponin elevation is associated with higher incidence of mechanical ventilation, cardiac function among those who required mechanical respiratory support remains largely unknown. In this study we demonstrate that 42% of the patients on mechanical respiratory support have reduced cardiac function. As shown in Figure 1 (panel C and D) an intubated young patient with a mildly elevated Troponin I of 0.15 ng/mL and acute respiratory distress syndrome had severe pulmonary hypertension and severely dilated RV with reduced function in the absence of pulmonary embolism. This case illustrates that acute pulmonary hypertension and RV failure can occur in COVID‐19 patients with severe hypoxia. In our series, pulmonary hypertension is seen in 60% of patients who required mechanical respiratory support. The prevalence is expected to be even higher considering pulmonary hypertension evaluation was only available in 41% of the entire cohort. Nevertheless, our findings suggest that cardiovascular abnormalities are important comorbidities in patients with respiratory failure. The critical role of cardiac function is further demonstrated among deceased patients in whom 43% had reduced cardiac function. Identification of reduced cardiac function may improve risk stratification for critically ill COVID‐19 patients. However, it remains to be determined if that knowledge will change clinical outcomes. As COVID‐19 is highly contagious it poses significant risk for staff performing the echocardiographic examinations. Under these circumstances, even a limited echocardiogram may yield highly valuable information, which may potentially change the course of patient management. Nonetheless, adequate personal protective equipment must be provided to the sonographers to ensure their safety.

The data from the current study should be interpreted within the context of several limitations. Bias is inevitable in selecting patients for an echocardiogram in this clinical cohort. Weighing the risk, clinicians are always mindful when ordering an echocardiogram for COVID‐19 patients. Therefore, patients who underwent the exam were more likely to have either a cardiac history or cardiac manifestations. Nevertheless, our findings are still highly relevant to the understanding of cardiac involvement in hospitalized COVID‐19 patients. In addition, due to lack of prior records, we are not able to fully differentiate prevalent from incident cardiac function abnormalities. Regardless, we found that the presence of reduced cardiac function has added vulnerability to COVID‐19 patients. It has also contributed significantly to the mortality of COVID‐19 patients. We recognize that the echocardiogram evaluation is likely incomplete in many cases especially for patients who were on mechanical ventilation which made the exam highly challenging. As a result, we may have underestimated the magnitude of cardiac abnormalities despite the high prevalence we have shown.

In conclusion, reduced cardiac function is common among hospitalized COVID‐19 patients with biomarkers of cardiac injury, those on mechanical respiratory support, and those deceased. It is also an independent predictor of mortality in addition to older age, elevated troponin and increased D‐dimer.

## Data Availability

Due to the nature of this research and our inability to completely remove personally identifiable information in compliance with HIPAA regulations, data presented here are not available for distribution to the public.
